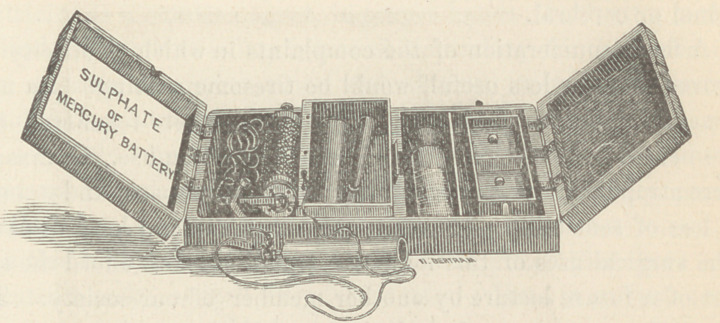# Medical Electricity

**Published:** 1881-11

**Authors:** S. V. Clevenger

**Affiliations:** First Vice President; Member of the American Electrical Society; of the American Association for the Advancement of Science; of the American Neurological Association; Trustee of the State Microscopical Society of Illinois; Physician to the South Side City Dispensary, etc., etc.


CHICAGO MEDICAL

JOURNAL & EXAMINER.

VOL. XLIII.—NOVEMBER, 1881.—NO. 5.

### Original Communications.

ARTICLE I.

MEDICAL ELECTRICITY. Opening Address before the Chicago Electrical Society, October 3, 1881. By First Vice President S. V. CLEVENGER, M.D., Member of the American Electrical Society; of the American Association for the Advancement of Science; of the American Neurological Association; Trustee of the State Microscopical Society of Illinois; Physician to the South Side City Dispensary, etc., etc.

THE PROBABLE NATURE OF ELECTRICITY has been so often discussed before this society, but little need be said under that head to-night. Sir Wm. Thompson stoutly affirms that "electricity in motion is heat;" Symmer and Du Fay regard it as consisting of two imponderable fluids permeating all ponderable matter (to my mind a most inconceivable hypothesis, as a *condition* is spoken of as a thing; a nonentity as a fluid); Tyndall, as an expounder of the laws of the correlation and conservation of forces, classifies it among the modes of motion, as a property of matter. A mental picture of energy in general may be made by conceiving *sound* to consist of motion of any substance with a rapidity between 16 and 40,000 vibrations per second, as the ear does not take cognizance of vibrations slower or faster than these; *light*, in round numbers, being constituted by rapid molecular movements numbering 450 billion at the red end of the spectrum, to 800 billion at the violet end; less rapid motions produce *heat*, while chemical effects are wrought by vibrations quicker than that of violet light in the invisible part of the spectrum. The atomic motions known as *electricity* are immensely more rapid than those of light, and even gravity is estimated by LaPlace to be produced by matter in motion, but inconceivably quicker than light or electrical motions, because matter is acted upon instantaneously through inter-planetary space; no calculation is possible of the time required for our sun to act upon the farthest planet. Not all the phenomena of electricity are accounted for by this assumption of a certain rate of vibration.

MAGNETISM can be regarded as an effect of electrization, and not as a distinct force by itself. It is in one sense a rectangular electricity, as it acts at right angles to the current causing it, and can no more be regarded as a separate energy than the attraction of gravitation can be considered apart from gravitation. Before Galvani's day the loadstone and artificial magnet were used in the treatment of disease. To-day we know the utter worthlessness of magnetism, pure and simple, as a therapeutic agent; and yet in our medical literature will be found occasional attempts at its resurrection. In surgery it has in a few instances proven useful, in aiding the extraction of foreign metallic substances from the body; as when a small piece of steel or iron had become embedded in the cornea, or had even penetrated to the lens.

I regard the advocacy of magnetism in headaches as deserving to rate with the magnetic jacket and insole nonsense we find advertised about the city.

Electrical shocks derived from the fish known as the torpedo were used ages ago as a cure for some forms of paralysis and rheumatism, but the beginning of electro-therapeutics was made 140 years since, by the suggestion of D'Helmstadt that electric sparks might be serviceable in treating diseases.

STATIC, FRANKLINIC OR FRICTIONAL ELECTRICITY was the first form to be used systematically in the cure of certain maladies.

Notwithstanding the advances made in physiological knowledge since static electricity was discovered, it has proven of but little use in medicine. Attempts have recently been made to resurrect it, and place it upon an equal footing with Galvanism and Faradization, but it does not seem probable that these attempts will succeed. Some of the great objections to the Holtz machine, used in generating static electricity, are its being cumbersome, expensive, and, even with the recent modifications, unreliable. Dr. A. D. Rockwell, an able electro-therapeutist of New York, in an article on this subject in the *Medical Record*, Sept. 17, 1881, acknowledges that the real *status* of Franklinic electricity requires more experiment and observation ; that the tonic and sedative effects obtained by Franklinization are not equal to those obtained by the use of dynamic electricity, and that in electro-surgery it is manifestly of but little value.

On the other hand, he claims that in a few instances pain may be relieved by means of this apparatus, where the other machines fail ; that in a few cases the treatment by sparks is preferred by the patients; that in old contractures, and possibly in cutaneous anæsthesia, Franklinization may possess some advantages. Bartholow, in his recent work on Medical Electricity, says very little about Franklinization. A few years ago an enthusiastic essay by Arthuis, of Paris, lauding static electricity as a cure-all, was translated into English in this country, but the work abounded with manifest errors, and was soon consigned to the limbo of worthless literature. At the last meeting of the American Neurological Society, held in New York city last June, quite a discussion was had over the probable value of Franklinic electricity in medicine. Opinions varied somewhat, but in the main it was considered as decidedly inferior to the two other forms. The methods of Charcot and Vigouroux in the use of a large platform, whereon numbers of patients were electrized at once, were characterized as unwarrantable. Dr. Morton described an apparatus, by means of which he claimed results similar to those obtained by the secondary coil, and erroneously called it a new induction current. Claims have been made that advantages are possessed by the current passing through Morton's interrupter, but this has been disputed, and Morton must experiment and report more fully before his apparatus will win favor. Dr. Onimus, one of the best electricians in Europe, refuses to use static electricity, claiming, after patient investigation, that it does no good, with the exception of affording relief temporarily in hysteria, sometimes; but this advantage not being one exclusively derivable from Franklinization, we can for the present pass over the Holtz machine and its improvements as a somewhat useless affair in treating diseases.

GALVANISM came into use among physicians at the beginning of this century, but it was coupled with mesmerism in the minds of the people, and falling into the hands of mountebanks, its actual worth was but little known until recently.

FARADIZATION came into vogue in 1831, and not until that date did electro-therapeutics assume a respectable appearance.

These two forms of electricity are essentially one and the same thing. An idea prevails, even among the educated, that there is an actual difference between them, as radical as between heat and light. Galvanic currents, by interruption with a suitable mechanism, and without the intervention of the secondary coil, can be converted into what will resemble in every respect the induced current. The Faradic is nothing more than a to-and-fro galvanic current. Its *effects*, however, as well as its uses in medicine, differ from those of the first named. This address not being intended as an elementary treatise upon electro-therapeutics, I shall merely set before you some of the cells used in generating the constant current. One of these, the celebrated Hill battery, is the invention of a member of this society, Dr. Hill, of this city.

The various forms of gravity batteries here shown are serviceable for offices, but the cells of Smee and Grenet are smaller and more portable. The Grove cup is powerful, but gives off objectionable fumes. The Leclanché element is coming into use for its permanency. As Leclanché cups of two ounces can be made, it is also portable, and being sealed, its serviceability is greatly enhanced. Dr. Hammond uses a modified Hill battery for office purposes.

SEALED, NO 1. COMPLETE

In all works on medical electricity, the Amperian method is used in designating current direction from the positive zinc plate through the solution to the negative copper plate, thence to the positive pole attached to the copper plate, completing the circuit through the wire to the negative pole attached to the positive zinc plate. This ideal direction of the current is actually not the true one. It can be readily shown that both the negative and the positive currents (*cæteris paribus*) travel at equal rates and in opposite directions. Thus, attach a galvanometer to each pole near the battery, and a third galvanometer at the junction of the two connecting wires; upon the circuit completion, the galvanometers nearest the poles will be instantaneously deflected, and the one at the junction subsequently. Designating the galvanometers in a circuit as A, B and C, if Ampere's hypothesis were true, the deflections would occur first at A, near the positive pole; next at B; then at C, next the negative pole. Instead of this we get Aand C simultaneously deflected, with B lastly.

The ingenuity of medical men has been taxed severely in constructing new forms of batteries (as the entire medical machine is styled), but the main points to be considered in a portable case are power, smallness, cleanliness and simplicity.

The tip cup of Kidder has some advantages, but the Smee and Leclanché, of smaller size, are preferable. Large sized cells are of no advantage over a number of small ones, for the law of Ohm proves that although the internal battery resistance is lessened by the large sized plates, the external resistance of the animal body is so very great that the quantity of electricity cannot be materially decreased unless a great number of cells be used. For the galvanic cautery, one or a few large cups are better than many small cells, the external resistance in this case being very small, as the current is only required to raise a small platinum wire to incandescence. Therefore the length of the time applied, and the number of cells used, represent the so-called dose of electricity. So at once we can see that the galvanometer, rheostat or resistance coils, are not of the slightest use in medical electricity, except for ornament, and the impression made upon the patient's mind by an array of mysterious implements.

The Galvano-Faradic Company, Dr. Kidder and Fleming and Talbot, make very good batteries, but as those of the Western Electric Company, of this city, have not been extensively figured in medical works heretofore, I exhibit that company's 32 cell

Galvanic and Chloride of Silver batteries. The first named has an electro-motive force of 60 Daniell cells; the chloride battery contains 16 or 24 cells, as desired.

This cautery battery produces a high electro-motive force with a single cell, the internal resistance of which is only .06 ohm. Four such cells as in this battery heat white hot 20 inches of No. 16 platinum wire.

A double cell Faradaic battery is here shown, with a secondary coil resistance of 150 ohms.

A single coil battery, as well as a large number of other forms, are made by the Western Electric Company, among which we find a diminutive affair called the Pocket Induction Machine, which works an hour without re-charging. Bisulphate of mercury is the solution used, and both currents are generated. Obstetricians will find this little affair invaluable in post partum hæmorrhage.

Except for surgical purposes and physiological experiment, the ordinary sponge electrodes answer the physician's purpose in most instances. A multitude of quite useless apparatus of this description has been invented, and in the majority of cases where electrodes are needed other than the sponge, they can be readily made of ordinary wire.

The Bell Induction Balance for detecting bullets, which gave unsatisfactory results in President Garfield's case, is described in the Philadelphia *Medical and Surgical Reporter* for August 13, 1881. It is a modification of the balance invented by Prof. E. D. Hughes, of London, England, a native of Bardstown, Ky. The first practical application of this instrument was to the detection of counterfeit money, as the several alloys affect the coils differently. Geo. Hopkins, in the *Scientific American*, July 30, 1881, describes a bullet hunter on Hughes' balance principle. The audiometer is another application, by Dr. Richardson, F.R.S., and I have been fortunate enough to secure one for exhibition. It is used for measuring the hearing, and enables the surgeon to record the progress of recovery from deafness (*vide* Report to Royal Society, *Medical Times and Gazette*, May 24, 1879).

The instrument consists of two primary coils, connected with Leclanché cells. Between these coils is a secondary coil sliding on a graduated bar. The distance between the primary and secondary coils registers the hearing capacity of the person to whose ear a telephone is applied. A microphone key interrupts the current, and the sound ceases to be heard at varying distances between the coils. The statement that an absolute zero of sound is obtainable, and the unnecessary graduation of the bar into millimeters, deserve criticism. It will prove useful to otologists. (Manufactured by Krohne & Sassmann, 8 Duke Street, Manchester Square, London, W.)

Medical electricity is divided into its applications to physiology, diagnosis, therapeutics and surgery. Galvani, Volta, Humboldt, Aldini, Du Bois-Raymond, Trowbridge, Pflüger, were prominent workers in the physiological field, and ponderous tomes have accumulated as the result of their researches. Though they added much to our knowledge, a careful revision of their labors is yet to be made in the light of a recent and better acquaintance with physiological laws. The most striking experiments in this connection were those of Fritsch and Hitzig upon the brains of dogs, and later, those of Ferrier upon that of the anthropoid ape. Definite movements of certain bodily parts took place by electrization of the bared brains of animals, and these discoveries tended more than anything else to disparage the so-called science of phrenology, establishing in its place what is known as the localization of function in the outer part of the brain. By electro-diagnosis, in many instances, we are enabled to locate the seat of disease, and determine whether it be muscular, nervous, spinal or cerebral.

A mere enumeration of the complaints in which electricity has proven more or less useful, would be tiresome reading. Its most general application has been to paralyses where regeneration of the nerves had occurred, and where, from non-use, the muscles were atrophied; for the relief of pain, to allay spasm and cramp; in loss of sensibility; in many constitutional and local diseases. The surgical uses of this force are numerous, but afford the subject of a future lecture by another member of our society. The unsatisfactory nature of applied electricity in medicine may be realized by inspection of the excellent work of the late Dr. Charles E. Morgan, a most sincere and able worker in this field. His facilities for gathering information on these topics were the very best, and his work, entitled Electro-Physiology and Therapeutics, contains 708 pages, of which only 25 are devoted to therapy. He concludes this small part of his large book with the words: "Such are the definite scientific applications of electricity to medical purposes; of the many others it need only be said that they are based on incorrect theory or diagnosis of disease, or an imperfect or incorrect knowledge of electro-physiology. I do not deny that *future* researches may enable us to do more, far more, than has hitherto been done in this direction."

Take the bulk of the respectable literature on this subject, and after excluding the ordinary descriptions of apparatus and their modes of action, it is astonishing how very little there is of a practical nature in electrical treatment of disease; yet we have electric baths and other elaborate apparatus, inviting the afflicted to take a shock at from five cents on the street corner, to $5 in some gorgeously furnished apartments. The simple little pocket battery will, in the hands of a regularly educated physician, prove infinitely more valuable in ailment treating than the most costly and imposing array of electrical apparatus, managed by an advertising ignoramus. Medical electricity does not yet deserve enough confidence in its curative properties to warrant any one becoming a specialist in that field, nor does the possession of a fine set of electrical apparatus ensure brains or education to its owner, any more than the ownership of an amputating set creates a surgeon.

I would not have it understood that I disparage the use of electricity as a curative agent, in proper hands; but owing to the medical applications being few, and depending for their efficacy upon an intimate acquaintance with disease, it stands to reason that a very ordinary instrument, in the charge of an intelligent and regularly educated physician, will be of some service where costly apparatus, under other circumstances, may easily prove hurtful.

In this connection it may as well be mentioned that there are educated quacks, as well as ignorant ones. Here is an instance: This great volume on Medical Electricity was written conjointly by two medical men in New York. The junior partner of the firm became disgusted with the trickeries of the senior, and has, since the dissolution of the partnership, published under his own name a small work, which embodies all the essentials of electrical treatment of disease. Since Dr. Geo. M. Beard has taken to lecturing on Trance, and has been proven guilty of attempting to impose upon medical men in England with some of his mesmeric exhibitions, it is safe to presume that to him alone may be credited the pointless, voluminous nonsense of the book alluded to, and Dr. Rockwell deserves credit for what there is good in it.

There is a disposition on the part of certain medical men to resort to innumerable tricks to increase their incomes, nor do we find this class peculiar to any one so-called school. Show, pretense, glitter, owlishness, pass current too often for knowledge and skill. Seeing this, otherwise excellent physicians are tempted to resort to the same charlatanry, excusing it on the ground of having to use psychological influences on their patient's behalf. They find their clients impressed by glittering machinery, and as the imagination undoubtedly has an influence over bodily conditions, why is it not justifiable to resort to measures so harmless, that good may result? Such Jesuitical reasoning is worthy of the highway robber. It is but another step to sugar-pill placebos, and trickery of the vilest sort, all in assumed interest of the patient.

Last winter I witnessed an electrical specialist applying the electrodes connected with a little voltaic pile to a case of acute rheumatism, and I felt perplexed when the patient said he experienced relief from pain after the application. In the first place, it is quite irrational to use electricity in this disease, and next, the current from the pile was of the weakest kind possible. The mystery was solved by the discovery that a few drops of a solution consisting of eight grains of atropine to the ounce of glycerine had been furtively applied to the painful muscles. A most powerful drug was used to deaden sensibility, while the effects were ascribed to the pile. This is psychological medicine with a vengeance.

A year or so ago, Neftel, of New York, who had published a work on the physiological effects of electricity applied to the head, was being extensively advertised by the *New York Observer* (a religious paper), as having effected marvelous cures of cataract with electricity. As Neftel had made no report of his cases to the medical journals, the genuineness of his cures was doubted, and the course of the *Observer* criticized. It was a disreputable advertising scheme.

To illustrate what the imagination will do in some cases, I shall conclude with an incident in my experience at Mercy Hospital about two years ago. A thoroughly educated gentleman, who had been a professor of chemistry in a college in this State, was suffering from spinal sclerosis, in consequence of which his arms and legs were drawn up against his body, and muscles had dwindled away seriously. Daily applications of electricity were made to special groups of muscles, after Duchenne's method, and concluded by application of the descending galvanic current to his spine. He never failed to respond to the soothing influence of the current, and every evening fell into a profound slumber after a few minutes' treatment in this way. Finally something happened to shake my belief in electricity as a hypnotic in all cases. After holding the wet sponges to his back as usual, patiently, and seeing that he had fallen asleep as he always did, I folded up the wires and turned to let down the cups from contact with the plates, and discovered that they had not been lifted at all. In fact, no current whatever had been passing. But I did not think it worth while to awaken him and tell him that he should not have gone to sleep.

## Figures and Tables

**Figure f1:**
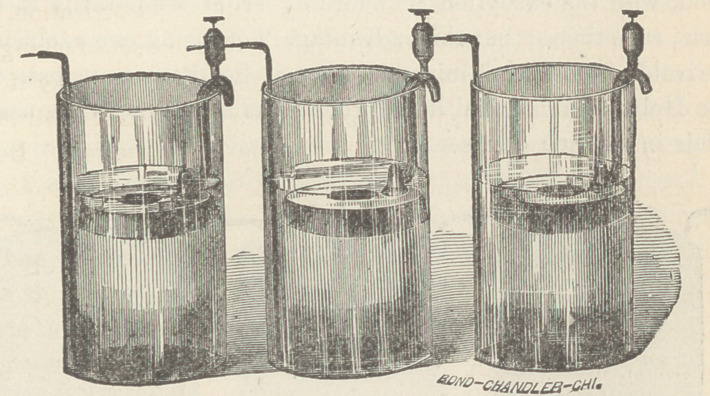


**Figure f2:**
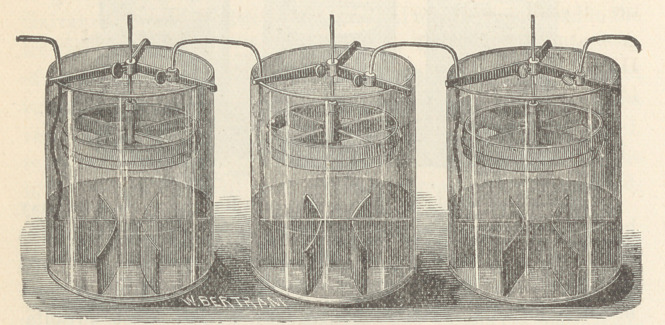


**Figure f3:**
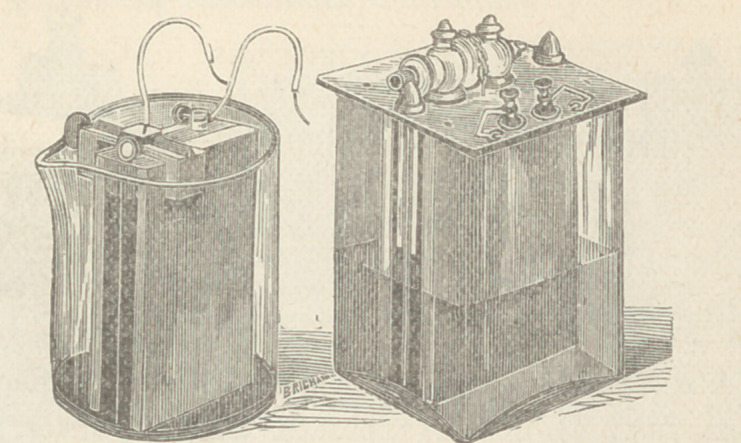


**Figure f4:**
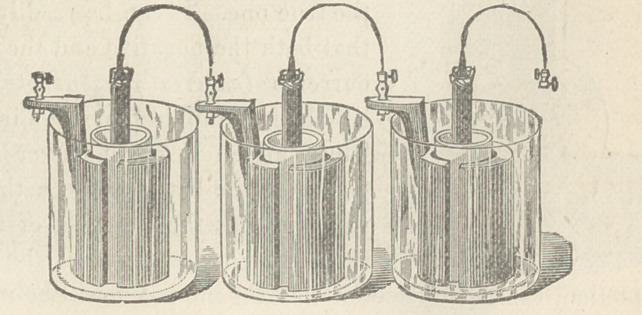


**Figure f5:**
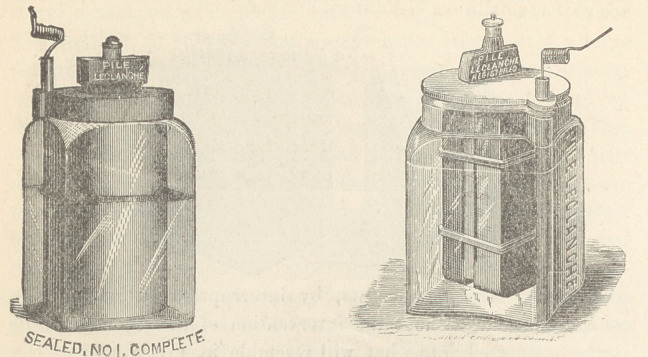


**Figure f6:**
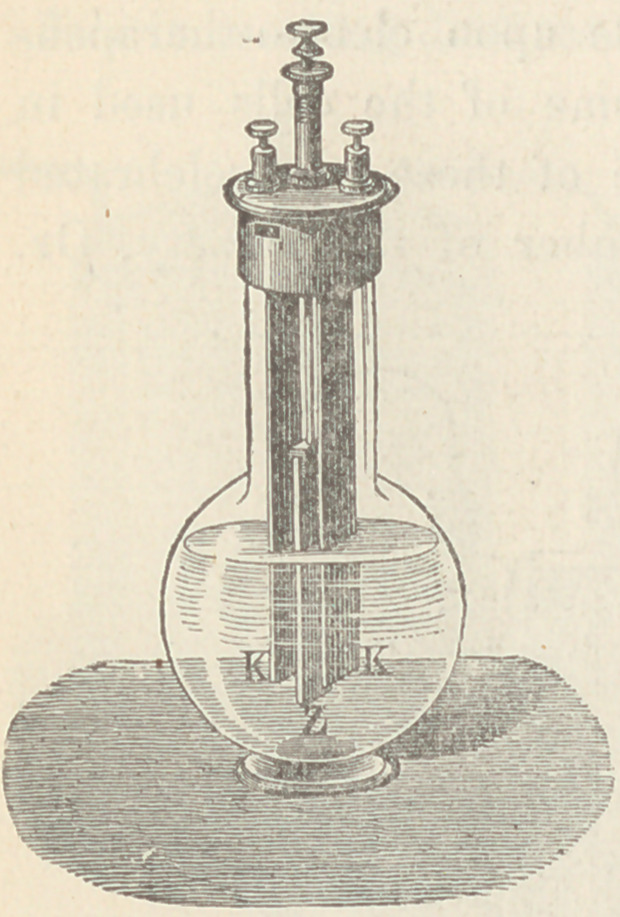


**Figure f7:**
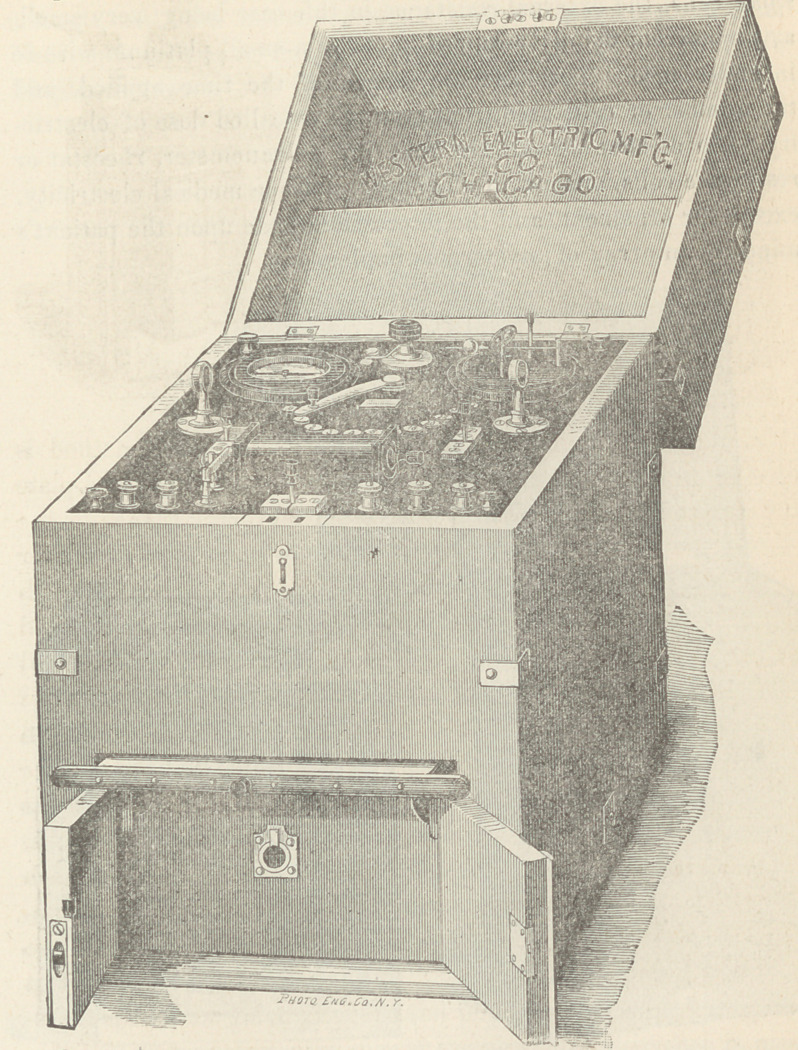


**Figure f8:**
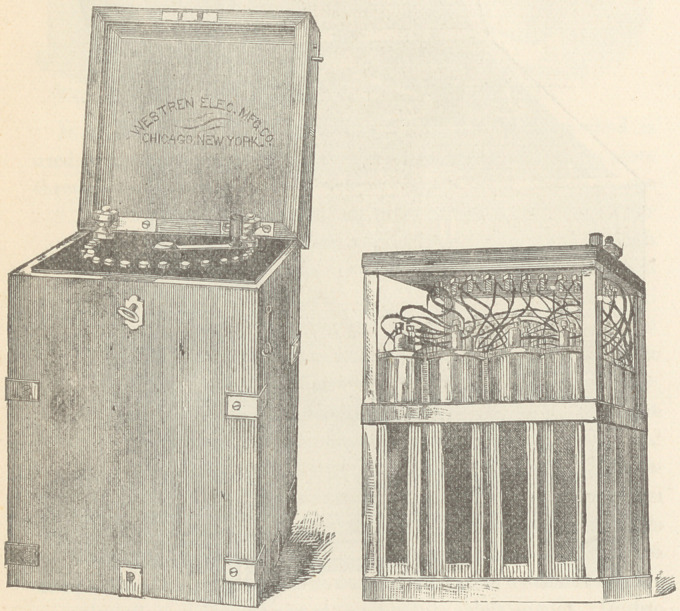


**Figure f9:**
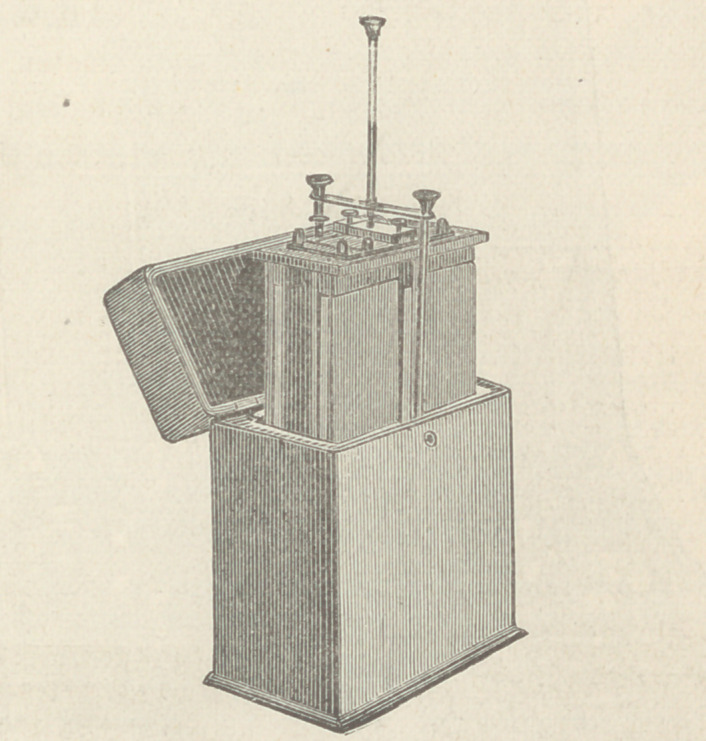


**Figure f10:**
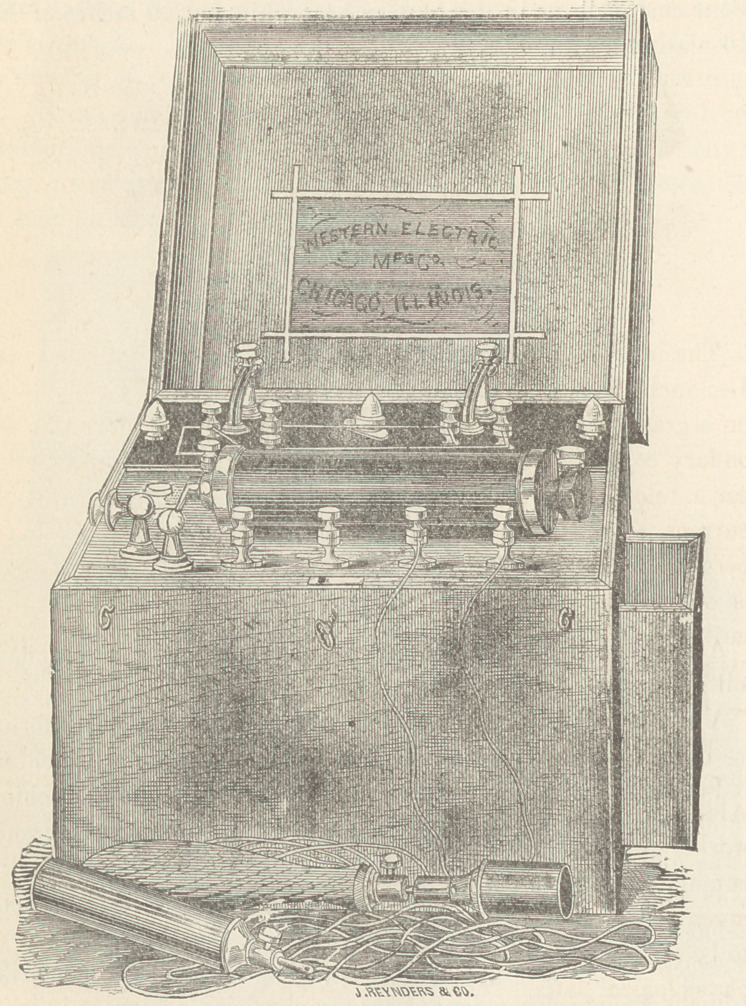


**Figure f11:**